# Comparison of intraoperative radiotherapy as a boost vs. simultaneously integrated boosts after breast-conserving therapy for breast cancer

**DOI:** 10.3389/fonc.2023.1210879

**Published:** 2023-06-20

**Authors:** Raluca Stoian, Jan-Philipp Harald Exner, Mark Gainey, Thalia Erbes, Eleni Gkika, Ilinca Popp, Simon K. B. Spohn, David Krug, Ingolf Juhasz-Böss, Anca-Ligia Grosu, Tanja Sprave

**Affiliations:** ^1^ Department of Radiation Oncology, University Hospital of Freiburg, Robert-Koch-Strasse, Freiburg, Germany; ^2^ German Cancer Consortium (DKTK) Partner Site Freiburg, German Cancer Research Center (dkfz), Neuenheimer Feld, Heidelberg, Germany; ^3^ Faculty of Medicine, University of Freiburg, Freiburg, Germany; ^4^ Department of Obstetrics and Gynecology, Medical Center, University of Freiburg, Freiburg, Germany; ^5^ Department of Radiation Oncology, University Hospital Schleswig-Holstein, Arnold-Heller-Str., Kiel, Germany

**Keywords:** breast cancer, intraoperative radiotherapy (IORT), SIB, toxicity, radiotherapy, adjuvant

## Abstract

**Background:**

Currently, there are no data from randomized trials on the use of intraoperative radiotherapy (IORT) as a tumor bed boost in women at high risk of local recurrence. The aim of this retrospective analysis was to compare the toxicity and oncological outcome of IORT or simultaneous integrated boost (SIB) with conventional external beam radiotherapy (WBI) after breast conserving surgery (BCS).

**Methods:**

Between 2009 and 2019, patients were treated with a single dose of 20 Gy IORT with 50 kV photons, followed by WBI 50 Gy in 25 or 40.05 in 15 fractions or WBI 50 Gy with SIB up to 58.80–61.60 Gy in 25–28 fractions. Toxicity was compared after propensity score matching. Overall survival (OS) and progression-free survival (PFS) were calculated using the Kaplan–Meier method.

**Results:**

A 1:1 propensity-score matching resulted in an IORT + WBI and SIB + WBI cohort of 60 patients, respectively. The median follow-up for IORT + WBI was 43.5 vs. 32 months in the SIB + WBI cohort. Most women had a pT1c tumor: IORT group 33 (55%) vs. 31 (51.7%) SIB group (p = 0.972). The luminal-B immunophenotype was most frequently diagnosed in the IORT group 43 (71.6%) vs. 35 (58.3%) in the SIB group (p = 0.283). The most reported acute adverse event in both groups was radiodermatitis. In the IORT cohort, radiodermatitis was grade 1: 23 (38.3%), grade 2: 26 (43.3%), and grade 3: 6 (10%) vs. SIB cohort grade 1: 3 (5.1%), grade 2: 21 (35%), and grade 3: 7 (11.6%) without a meaningful difference (p = 0.309). Fatigue occurred more frequently in the IORT group (grade 1: 21.7% vs. 6.7%; p = 0.041). In addition, intramammary lymphedema grade 1 occurred significantly more often in the IORT group (11.7% vs. 1.7%; p = 0.026). Both groups showed comparable late toxicity. The 3- and 5-year local control (LC) rates were each 98% in the SIB group vs. 98% and 93% in the IORT group (LS: log rank p = 0.717).

**Conclusion:**

Tumor bed boost using IORT and SIB techniques after BCS shows excellent local control and comparable late toxicity, while IORT application exhibits a moderate increase in acute toxicity. These data should be validated by the expected publication of the prospective randomized TARGIT-B study.

## Introduction

Women receiving breast-conserving surgery (BSC) benefit from radiotherapy (RT) of the whole breast (WBI) and, in the case of risk factors for local recurrence, also from a dose escalation in the tumor cavity (1–3).

External beam RT is the most frequent method used for adjuvant treatment of the whole mammary gland and boost irradiation (4). Although boost irradiation was established with sequential administration, dose escalation in the tumor bed is often performed using a simultaneous integrated boost (SIB) (4). Recently reported data from randomized trials comparing conventionally fractionated WBI with a SIB versus sequential boost yielded comparable oncological outcomes and favorable acute toxicity and quality of life (5–9).

Alternatively, booster irradiation of the tumor bed can be achieved using intraoperative RT (IORT) (10–13). IORT is delivered with electrons (IOERT) or 50 kV photons as a single fraction during surgery as a “same-day approach.” Since the SIB concept has reduced the overall treatment time by about five to eight fractions, the potential advantages of IORT application are confined to geographic and temporal limitations.

A persistent challenge for SIB applications is the geometric accuracy of tumor bed cavity localization to prevent local recurrence. To reduce geometric target misses after oncoplastic reconstruction, the tumor bed is marked. Most typically, tumor borders are marked with surgical titanium clips (14), whereas interstitial markers tend to mark the surrounding tissue. However, a recent study points to the occurrence of a clinically meaningful change in the location of fiducials in relation to the tumor cavity that occurs between treatment planning and start (15). By contrast, during IORT, the surgical tumor bed is visualized, and thus both boost volume and skin dose can potentially be reduced.

The surgical fluid can stimulate the proliferation of tumor cells. Analyses of wound fluid from women treated with high-dose IORT suggest that IORT has a positive effect on the tumor microenvironment (16, 17). Perhaps an additive benefit of IORT for local control can be expected.

Data from the randomized TARGIT-B trial evaluating IORT as a boost vs. SIB are still pending. Thus, the aim of this retrospective study was to compare the oncological outcomes and toxicities of IORT boost and SIB in early breast cancer.

## Materials and methods

Women treated with IORT as a tumor bed boost or SIB with WBI from 2009 to 2019 at the University Hospital of Freiburg were retrospectively included in this study. Preliminary results on the use of IORT as an anticipated boost in a large population have previously been published separately (13). Institutional criteria for selecting women at high risk of recurrence for IORT boost or SIB include patients’ premenopausal status or postmenopausal status with additional risk factors such as tumor size ≥2 cm, extensive intraductal component, G3, HER2-positive, or triple-negative breast cancer (TNBC). Approximately 30% of breast cancer patients in our clinic receive a boost indication.

BCS with sentinel lymph node excision or axillary nodal dissection was completed according to institutional protocols. Systemic therapy was performed according to current guidelines and the recommendations of the interdisciplinary oncology panel.

A single IORT dose of 20 Gy was prescribed to the applicator surface (range 20–50 mm) and skin-sparing was delivered using 50-kV X-rays with the INTRABEAM miniature X-ray generator (Carl Zeiss Surgical, Oberkochen, Germany). Subsequently, WBI was applied using conventional fractionation (50–50.4 Gy in 25–28 fractions) or hypofractionation (40.05 Gy in 15 fractions). SIB was delivered up to 58.80–61.60 Gy in 25–28 fractions. CT-based (Brilliance, CT Big Bore, Philips, Cleveland, OH) three-dimensional treatment planning (Oncentra MasterPlan, Nucletron, Veenendaal, The Netherlands, or Eclipse™ planning systems (Varian Medical Systems)) was performed using tangential portals (6 or 18 MV; Synergy; Elekta, Crawley, United Kingdom). Women with left-sided breast cancer received WBI in the deep inspirational breath hold (DIBH) technique with surface-guided RT (C-RAD, Catalyst, C-RAD AB, Uppsala, Sweden). In a few cases, intensity-modulated RT (IMRT) or volumetric modulated arc therapy (VMAT) were used to reduce lung and heart doses.

Breast ultrasound was done every 6 months for the first three years. Mammograms were performed six months after RT and annually after the first mammogram. Recurrence was confirmed by biopsy.

All women were monitored every three to six months for the first two years, followed by annual visits afterwards. Adverse side effects within three months after RT were classified as acute, whereas any events occurring beyond three months after the end of RT were categorized as late toxicities. Acute side effects were evaluated according to the Common Terminology Criteria for Adverse Events version 5.0. Late toxicity was assessed based on modified Late Effects in Normal Tissues criteria (subjective, objective, management, and analytic, LENT-SOMA). The cosmetic results were not recorded.

### Statistical analysis

Outcomes included local control rate (LC), progression-free survival (PFS), and overall survival (OS). All were defined from the date of IORT for the IORT + WBI group or the start of RT for the SIB + WBI group to the pertinent event. Survival times were calculated using the Kaplan–Meier method. Dates are reported as mean, median (range), and frequency.

A propensity score matching analysis was performed with a logistic regression that considered the following: age, postoperative tumor size and status of the regional lymph node, tumor grading, and immunophenotype. *P*-values < 0.05 were considered statistically significant. Statistics were performed with SPSS version 29 (IBM, Armonk, NY, USA).

## Results

**Figure 1:**
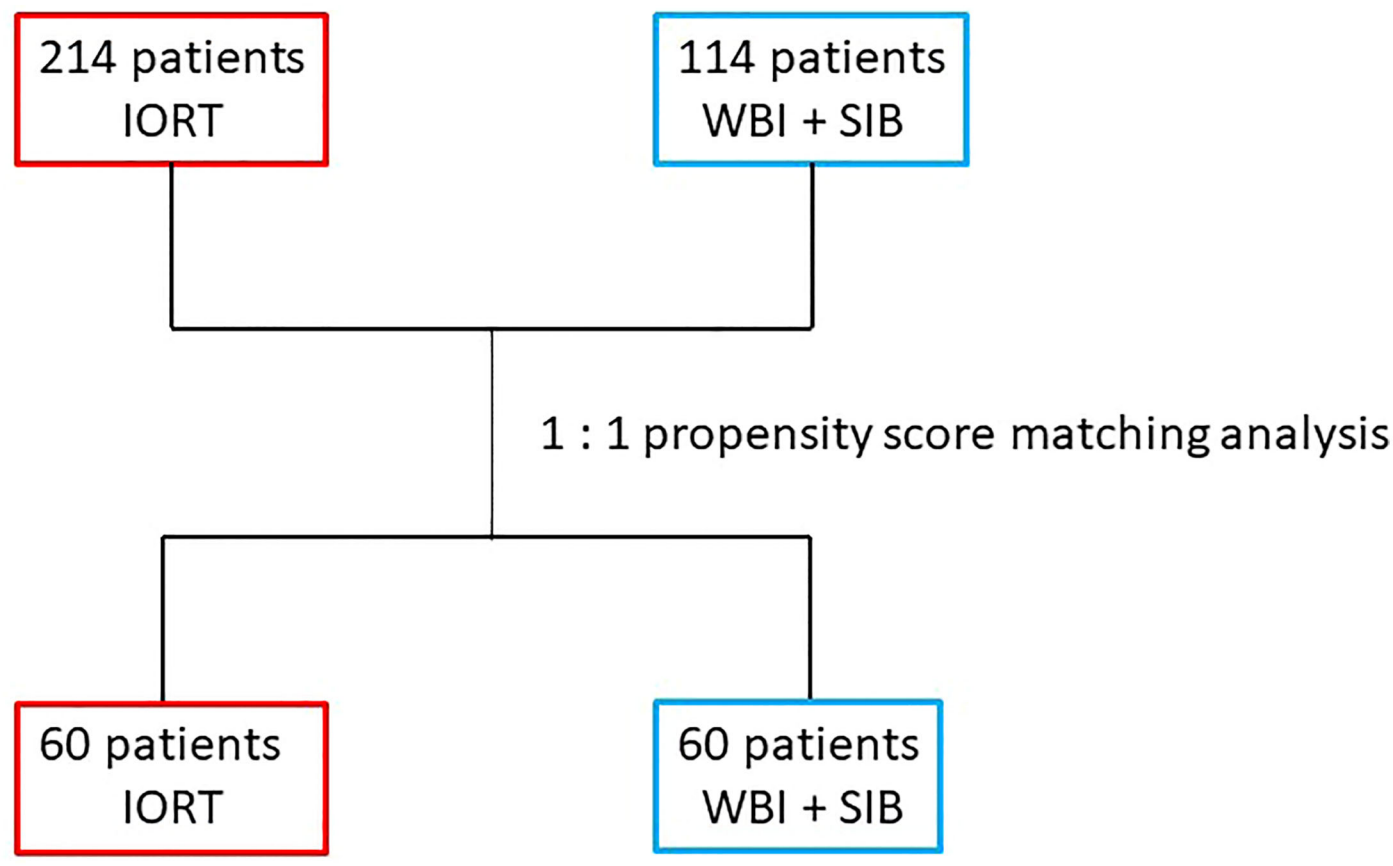
Flow chart.

A total of 214 women treated with IORT + WBI and 114 women receiving RT using SIB + WBI were identified and included in this analysis (**Figure 1**). After 1:1 propensity score matching, 60 patients remained in each group. After propensity score matching, treatment groups were well balanced (**Table 1**). The median age was 56.2 years (range 30–82) in the IORT + WBI group versus 58 years (range 40–85) in the SIB + WBI group. Most women in both groups were postmenopausal. Most patients had T1 disease: in the IORT + WBI group 39 (65%) vs. 38 (63.3%) in the SIB + WBI group. Most women were clinically node negative. Only a few women had high-grade disease: 10 (10.7%) in the IORT + WBI cohort vs. 9 (15%) in the SIB + WBI cohort. Two women (3.3%) in the IORT + WBI vs. eight women (13.3%) in the SIB + WBI had a triple-negative disease.

**Table 1:** Patient and tumor characteristics.

	IORT+WBI	SIB+WBI	
*Total n= 120 patients*	*n (%)*	*n (%)*	*p-value*
	60	60	
Median age in years (range)	56.2 (30-82)	58 (40-85)	0,153^a^
Postmenopausal	36 (60)	44 (73.3)	0,121^b^
***Side***			
Right	29 (48.3)	28 (46.7)	0,854^b^
Left	31 (51.7)	32 (53.3)	0,854^b^
***Tumor stage***	*** ***	*** ***	
1a	3 (5)	3 (5)	0,972^b^
1b	3 (5)	4 (6,6)	
1c	33 (55)	31 (51,7)	
2	21 (35)	22 (36,7)	
***Nodal stage***	*** ***	*** ***	
0	44 (73,4)	45 (75)	0,641^b^
mi	2 (3.3)	4 (6.7)	
1a	12 (20)	8 (13.3)	
2a	2 (3.3)	3 (5)	
***Grading***			
G1	7 (11.7)	5 (8.3)	0,921^b^
G2	42 (75.9)	45 (75)	
G3	10 (10.7)	9 (15)	
unknown	1 (1.7)	1 (1.7)	
***Immunophenotype***			
Luminal A	12 (20)	15 (25)	0,283^b^
Luminal B	43 (71,6)	35 (58.3)	
TNBC	2 (3.3)	8 (13.3)	
HR status: positive	56 (93.3)	52 (86.7)	
Her2neu status: positive	3 (5)	2 (3.3)	

Patient and tumor characteristics consisting patients treated by IORT+WBI and SIB+WBI in our institution between 2009 and 2019 (n = 120, 60 women in each group). Staging of breast cancer was based on the 7th Edition of the UICC TNM classification.

IORT, intraoperative radiotherapy; HR, hormone receptor positive; TNBC, triple negative breast cancer; p, pathological; SIB, simultaneous integrated boost; WBI, whole breast irradiation.

a Paired t-Test.

b Chi quadrat.

However, when treatment characteristics were analyzed, there were statistically significant imbalances in the distribution of resection margins, neoadjuvant chemotherapy, frequency of conventionally fractionated WBI, and use of the DIBH technique (**Table 2**). In the SIB + WBI cohort, more women (18, 30%) received re-resection vs. IORT + WBI (7, 11.7%) (p = 0.013). The abovementioned re-resection for positive margins was performed as part of the first surgery in all patients. After re-resection, all tumor cavity margins were clear. Neoadjuvant chemotherapy was also applied more frequently in the SIB + WBI group (35% vs. 6.7%; p = 0.0001). In addition, fewer women in the IORT + WBI group received conventionally fractionated RT (90% vs. 100%; p = 0.021). Only five women (8.3%) received hypofractionated WBI after IORT. The DIBH technique was also used less frequently in the IORT + WBI group (13.3% vs. 40%; p = 0.009). A total of 16 (26.6%) and 10 (16.6%) women underwent adjuvant chemotherapy in the IORT + WBI and SIB + WBI cohorts, respectively. Most women in each group received endocrine therapy. A single dose of 20 Gy IORT was successfully applied to all patients using an applicator surface median of 35 mm (range 20–50 mm). The median time between IORT and WBI was 59 days (range 28–228).

**Table 2:** Treatment characteristics.

	IORT + WBI	SIB + WBI		
*Total n= 120 patients*	*n (%)*	*n (%)*	*p-value*	
BCS	60 (100)	60 (100)		
SLND	56 (93.3)	58 (96.7)	0.402^b^	
ALND	5 (8.3)	4 (6.7)	0.728^b^	
Resection status				
R0	52 (86.7)	45 (75)	0.813^b^
R1	6 (10)	6 (10)	
Re-resection needed due to R+ status	7 (11.7)	18 (30)	0.013^b^
Neoadjuvant chemotherapy	4 (6.7)	21 (35)	0.0001^b^
Adjuvant chemotherapy	16 (26.6)	10 (16.6)	0.183^b^
*Endocrine therapy*	56 (93.3)	52 (86.7)	0.223^b^
simultaneous	3 (5)	4 (6.7)	0.848^b^
adjuvant	13 (21.7)	13 (21.7)	
upfront	40 (66.7)	35 (58.3)	
SIB Dose				
2.2 Gy		16 (26.7)	
2.4 Gy		44 (73.3)	
*IORT Dose*			
20 Gy	60 (100)		
Applicator surface median (mm)	35		
WBI				
normo-fractionated (25–28×)	54 (90)	60 (100)	0.021^b^
hypo-fractionated (15×)	5 (8.3)	0	
3DRT	54 (90)	52 (87.6)	0.395^b^
IMRT/VMAT	5 (8.3)	8 (13.3)	
DIBH	8 (13.3)	24 (40)	0.009^b^
Regional nodal irradiation				
normo-fractionated (28×)	0	4 (6.7)	

ALND, axillary lymph node dissection; BCS, breast conserving surgery; DIBH, deep inspiration breath hold technique; 3DRT, 3D-conformal radiotherapy; Gy, gray; IMRT, intensity modulated radiotherapy; IORT, intraoperative radiotherapy; SLND, sentinel lymph node dissection; VMAT, volumetric modulated arc therapy; WBI, whole breast irradiation.

a Paired-t-test.

b Chi-squared test.

Treatment details for radiotherapy using IORT + WBI and SIB + WBI of breast cancer patients (n = 120, 60 women in each group).

WBI was applied using standard tangential treatment portals in most cases in the IORT + WBI cohort 54 (90%) vs. 52 (87.6%) in the SIB + WBI cohort. Only five patients (8.3%) received WBI using IMRT/VMAT in the IORT + WBI vs. eight (13.3%) women in the SIB + WBI group. Only one woman did not receive WBI. She has explicitly renounced WBI at her own request, while guaranteeing close clinical follow-up and adjuvant systemic therapy. Four women (6.7%) in the SIB + WBI cohort received conventionally fractionated RT to the supraclavicular fossa with 50.4 Gy in 28 fractions.

**Table 3** displays the toxicity profile of the study population. No women experienced a grade 4 event. Grade 3 acute radiation dermatitis occurred in six (10%) women in the IORT + WBI cohort and seven (11.6%) in the SIB + WBI group. In the IORT + WBI group, significantly more women reported fatigue (grade 1: 21.7% vs. 6.7%; grade 2: 0% vs. 1.7%; p = 0.041). In the IORT + WBI group, women had a numerically higher incidence of pain. Lymphedema grade 1 of the breast was recorded more frequently in the IORT + WBI group (11.7% vs. 1.7%; p = 0.026). Late toxicities were almost exclusively grade 1 toxicities, with just one case of radiodermatitis grade 2 after SIB + WBI.

**Table 3:** Toxicity.

	IORT + WBI				SIB				
	Toxicity grade n (%)								
	0	1	2	3	0	1	2	3	p-value
Acute toxicity post WBI									
Dermatitis	4 (6.6)	23 (38.3)	26 (43.3)	6 (10)	1 (1.7)	3 (5.1)	21 (35)	7 (11.6)	0.309^b^
Seroma/hematoma breast	53 (88.3)	7 (11.7)			58 (96.7)	2 (3.3)			0.078^b^
Seroma/hematoma axilla	59 (98.3)	1 (1.7)			60 (100)				0.315^b^
Wound infection	59 (98.3)	1 (1.7)			60 (100)				0.315^b^
Wound dehiscence	59 (98.3)	1 (1,7)			60(100)				0.315^b^
Fatigue	47 (78.3)	13 (21.7)			55 (91.7)	4 (6.7)	1 (1.7)		0.041^b^
Pain	47 (78.3)	13 (21.7)			49 (81.6)	10 (16.7)	1 (1.7)		0.488^b^
Lymphodema	53 (88.3)	7 (11.7)			59 (98.3)	1 (1.7)			0.026^b^
Late toxicity post WBI									
Dermatitis	57 (95)	3 (5)			59 (98.3)		1 (1.7)		0.133^b^
Seroma/hematoma breast	54 (90)	6 (10)			58 (96.7)	2 (3.3)			0.143^b^
Seroma/hematoma axilla	60 (100)				60 (100)				
Wound infection	60 (100)				60 (100)				
Fatigue	54 (90)	6 (10)			50 (83.3)	10 (16.7)			0.283^b^
Pain	51 (85)	9 (15)			51 (85)	9 (15)			1^b^
Lymphodema	53 (88.3)	7 (11.7)			51 (85)	9 (15)			0.591^b^

IORT, intraoperative radiotherapy; WBI, whole breast irradiation.

b Chi-squared test.

Acute and chronic radiotherapy-related toxicities after IORT + WBI and SIB + WBI according to the Common Terminology Criteria for Adverse vents (CTCAE v5.0).

**Figure 2:**
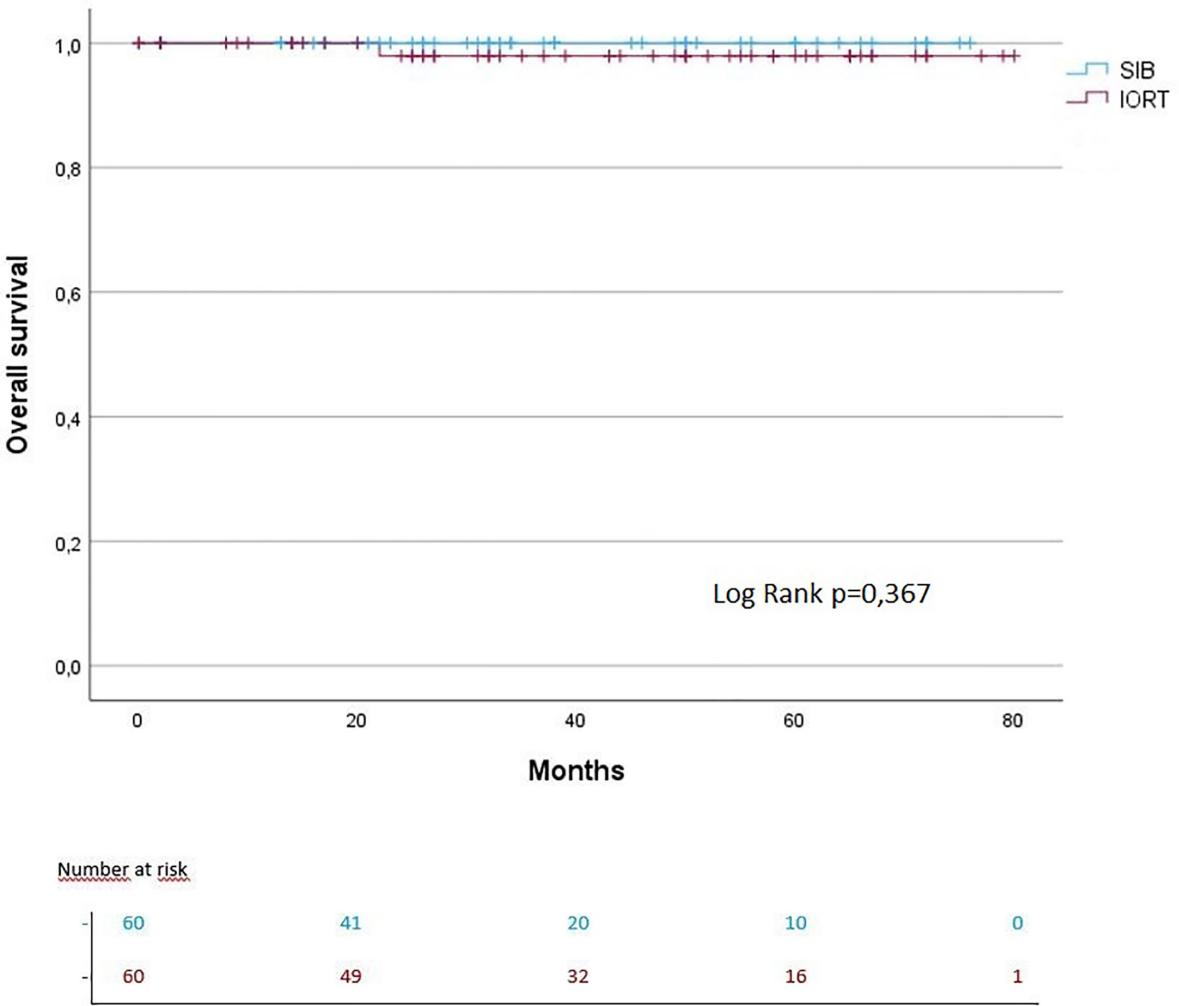
Kaplan–Meier curves regarding OS.

**Figure 3:**
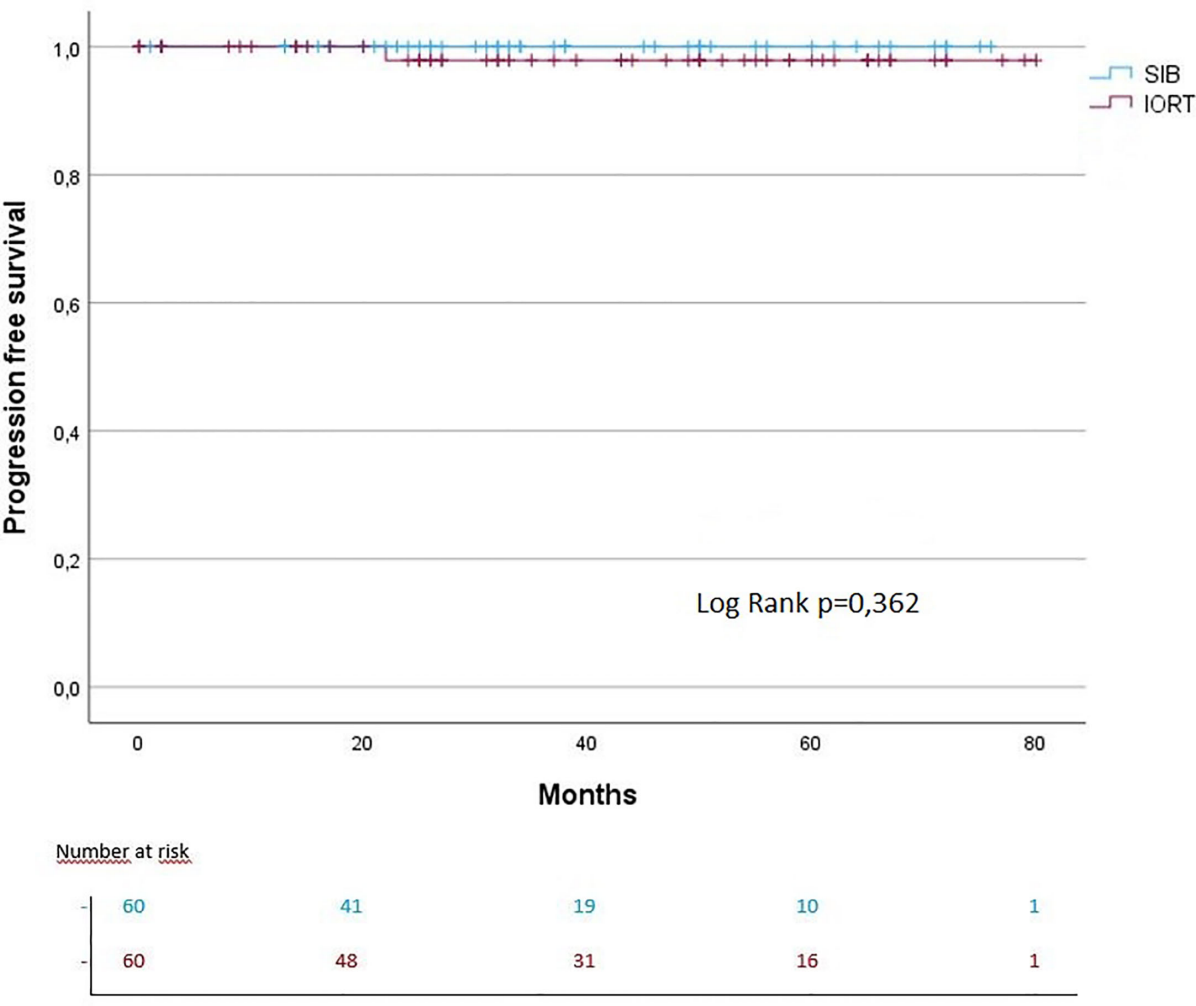
Kaplan–Meier curves regarding PFS.

**Figure 4:**
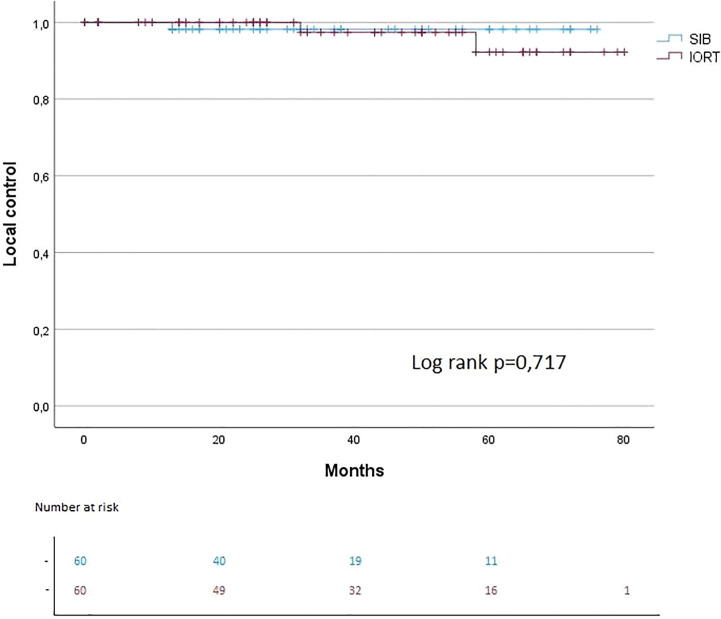
Kaplan–Meier curves regarding LC.

The median follow-up for IORT + WBI was 43.5 months vs. 32 months in the SIB + WBI cohort. The 3- and 5-year OS were each 97% in the IORT group vs. 100% in the SIB group (OS: log rank p = 0.367). The 3- and 5-year PFS were each 100% in the IORT group vs. 100% in the SIB group (PFS: log rank p = 0.362) (**Figures 2**, **3**). The 3- and 5-year local control (LC) rates were each 98% in the SIB group vs. 98% and 93% in the IORT group (LS: log rank p = 0.717) (**Figure 4**). All recurrences were in the boost region (in-field recurrences).

## Discussion

As far as we know, this is the first propensity score matching study to evaluate the outcome of women with breast cancer who had IORT with photons as an anticipated boost compared to conventionally fractionated WBI with SIB. This single-institutional retrospective study demonstrates early results from IORT boost with 50 kV photons regarding comparable oncological outcomes and mild late toxicity. After a short follow-up, OS and PFS did not differ between the groups.

Currently, the main source of evidence for boosting IOERT before WBI comes from pooled cohort analyses performed by the European group of the International Society for Intraoperative Radiotherapy (ISIORT Europe) (18). The long-term results, especially in high-risk patients, are persuasive, with excellent tumor control rates of 95%. This was achieved by avoiding the geographic and temporal misses coupled with the radiobiological superiority of the high single doses. IOERT spared the skin and produced a favorable cosmetic outcome (11, 18).

In this study, slightly more acute side effects occurred with IORT compared to external beam radiotherapy alone. Only the incidence of acute intramammary lymphedema and fatigue were significantly higher in the IORT + WBI group (**Table 3**), whereas long-term toxicity was comparable in the IORT + WBI groups (**Table 3**). Perhaps the clinical follow-up was better documented specifically for the women with IORT boost compared to women with SIB + WBI. This could make the acute adverse event rate appear exaggerated in the IORT + WBI group. A recent 10-year analysis of the use of IOERT boost (12 Gy) and hypofractionated WBI in young women ≤48 years old by Leonardi et al. reported excellent local control (19). The authors observed a 10-year cumulative rate of local recurrence of 4.1% and OS of 96.5% (19). Our study achieved similar oncological results, considering the significantly shorter follow-up. Chronic lymphedema by Leonardi et al. was reported as grade 1 in 17.2% and grade 2 in 2.5% of patients. Our study recorded slightly lower values for chronic lymphedema grade 1 in both arms (**Table 3**). Recently, 10-year results of an Italian phase 3 non-inferiority trial were published, which investigated IOERT with 10 Gy vs. sequential external beam boost with 10 Gy in five fractions and after WBI with 50 Gy in 25 fractions, respectively (20). High local control rates without significant differences were observed in both groups (20). In the IOERT group at five and ten years, the risk of local recurrence was 0.8% and 4.3%, respectively, whereas in the external beam boost arm, the rates were 4.2% and 5.3%, respectively. Remarkably, after 10 Gy IOERT, local recurrence occurred a median of four years later compared to sequential boost. Furthermore, the authors report acute postoperative seroma after IOERT in seven women (5.6%) and wound healing disorders in three women (2.4%) (20). In our analysis of the IORT arm, similar rates of postoperative seroma and wound dehiscence were observed.

Currently, there are still few reports on the use of IORT 1 × 20 Gy as an anticipated tumor bed boost. The long-term outcomes by Pez et al. in a large collective with IORT Boost and WBI showed a low locoregional recurrence rate and few serious adverse events, mainly fibrosis and pain (10). Notably, the occurrence of higher-grade fibrosis after a median of 3 years can be considered a consequence of a possibly too short interval between IORT and WBI (21). Due to the short follow-up interval in our study for the IORT + WBI groups, late toxicity may be underreported.

The oncoplastic reconstruction approach during BCS improves the cosmetic outcome but challenges clear delineation of the tumor bed (22–24). IO(E)RT of the visualized tumor cavity prior to reconstruction avoids geographic misses and thereby reduces the boost volume. Based on numerous randomized clinical trials with RT alone, a low α/β value of 3.5–4 was adopted for breast cancer (25–28). Thus, according to EQD_2Gy_, the IORT boost of 1 × 20 Gy results in a prescribed dose of 80 Gy on the applicator surface and approximately 5 Gy and EQD2 7.5 Gy at 1 cm depth. Consequently, according to EQD_2 Gy_, after IORT and conventionally fractionated WBI, 130 Gy were applied at the rim of the tumor cavity and 57.5 Gy at a depth of 1 cm. There was a lower dose according to EQD_2Gy_ with 124 Gy at the rim of the tumor cavity and 52 Gy at a depth of 1 cm after hypofractionated WBI. Including the time factor between IORT and adjuvant WBI in the above calculation would lead to a reduction in the isoeffective dose. Given the wide time range between IORT and WBI in our collective, this calculation does not appear to be usefully applicable.

Correspondingly, 64 Gy were applied in the SIB + WBI group according to EQD2 after SIB with 2.4 Gy in the boost volume during conventionally fractionated WBI. Moreover, by definition, the SIB volume was significantly larger than the tumor bed exposed during IORT.

Targeted dose escalation in the tumor bed up to 66 Gy (WBI 50 Gy and 16 Gy sequential boost) using external beam RT was investigated in the randomized EORTC “boost versus no boost” trial (1). After a median follow-up time of about 5 years, a clear benefit of dose escalation on local control was demonstrated, which was confirmed at 20 years in young patients (1, 3). However, excellent local control was achieved at the cost of higher rates of local fibrosis (3). Additional dose escalation in the tumor bed was investigated in the Young Boost trial in young women under 50 years old (29). The local recurrence risk and cosmetic outcome were compared between the 26 Gy and 16 Gy boost dose groups. Significantly more higher-grade fibrosis was observed in the dose-escalated arm. In addition to dose escalation, SIB administration was also identified as a risk factor for fibrosis development (29).

SIB is the best-established approach to reducing the total treatment time. The results were published from three randomized trials that compared the use of SIB versus sequential boost (6, 8, 30). The oncological efficacy was equivalent, with less acute toxicity in favor of the SIB approach (30). Krug et al. recorded acute toxicity in the IMRT-MC2 phase 3 trial for the SIB arm as follows: at the RT end, grade 2 radiation dermatitis was 29.1%, grade 3 was 3.5%, and at the first follow-up, grade 2 was 0.5% (8). In our study in the SIB + WBI arm, comparable acute grade 2 radiodermatitis and a slightly higher number at grade 3 were observed (**Table 3**). In IMRT-MC2 in the SIB arm, grades 1 and 2 pain was 13.2 and 1.8%, respectively, at the RT end, and at first follow-up, 24.7% for grade 1 and 0.9% for grade 2 events (8). In our study, similar values for pain grades 1: 10 (16.7%) and 2: 1 (1.7%) were recorded (**Table 3**).

Hypofractionation in the treatment of stage I and II breast cancer is the standard. Currently, the combination of hypofractionation and IOERT Boost is being investigated in the HIOB study. The HIOB trial reported excellent acute and late toxicity after 3 years of follow-up (12).

Despite these promising results, the limitations of this study should be addressed. This retrospective study, which was based on propensity score matching, was conducted in only one institution. Patients were carefully selected for IORT, whereas the women from the SIB + WBI group often received surgical treatment outside our institution. This limits the transferability to other patient groups. A major cause of bias may be our selection of prognostic factors for propensity score matching. Although this resulted in a balanced group distribution in demographic data and patient characteristics, significant differences were obtained when considering therapy and tumor characteristics. The considerable disparity between groups in terms of neoadjuvant chemotherapy may have an impact on early and late toxicity. In particular, the significant aggravation of acute toxicity in the IORT + WBI arm needs to be critically questioned. The short follow-up time in our cohort only provides limited information on toxicity events and progression-free survival that occur over the long term.

Finally, there is mature data for the use of VMAT for SIB + WBI applications (31, 32). The VMAT technique improves dose homogeneity, cosmetic outcome, and risk of organ sparing. However, the accepted compromise for this improved dose homogeneity is increased low-dose exposure to surrounding organs. The clinical relevance of low doses in secondary carcinogenesis remains a field of investigation (33, 34). In contrast, during IORT application, the surrounding tissue is optimally spared (35). Perhaps the long-term data from the IORT application can provide a further clinical advantage in reducing secondary carcinogenesis and non-breast cancer mortality (36).

In summary, tumor bed boost using IORT and SIB techniques after BCS showed excellent local control and comparable late toxicity. However, IORT was associated with a moderate increase in acute toxicity. These data should be validated by the expected publication of the prospective randomized TARGIT-B study.

## Data availability statement

The raw data supporting the conclusions of this article will be made available by the authors, without undue reservation.

## Ethics statement

The studies involving human participants were reviewed and approved by the Ethics Committee of the University Hospital Freiburg. Written informed consent for participation was not required for this study in accordance with the national legislation and the institutional requirements.

## Author contributions

TS and RS: Study conception and study design. RS, J-PE, SS, IP, and TS: Data acquisition, data analysis and data interpretation. TE, EG, and MG performed the IORT. TS, RS, and IP: Statistical analysis. TS, RS, and DK: Manuscript editing. RS, J-PE, MG, TE, EG, DK, IP, SS, IJ-B, A-LG, and TS: Manuscript review. All authors contributed to the article and approved the submitted version.
